# Jefferson Medical College of Philadelphia

**Published:** 1844-03-23

**Authors:** R. M. Huston

**Affiliations:** Dean of the Faculty


### JEFFERSON MEDICAL COLLEGE

### OF PHILADELPHIA.

At a Public Commencement, held on the 20th of March, 1844, the Degree of Doctor of Medicine was conferred on the following gentlemen, by the Rev'd ASHBEL GREEN, D. D., L. L. D., President of the College ; after which a Valedictory Address was delivered by Professor MÜTTER.

*Maine.*

| | Subject of Theses. |
|---|---|
| Samuel W. Blanchard, | De Natura Mulieris. |
| Frederick Robie, | Water, its properties and effects. |
| Franklin Scammon, | Measles. |
| Edward W. Southwick, | Relations of Chemistry to Physiology. |

*New Hampshire.*

| | |
|---|---|
| Simeon Sessions Dana, | Influence of the atmospheric air on human health. |
| John D. Ford, | Nature and causes of Tracheitis. |

*Massachusetts.*

| | |
|---|---|
| Silas S. Brooks, | Diseases of the lachrymal organs. |
| John M. Harlow, | Counter-Irritation. |
| Joseph H. Haskell, | Cholera Infantum. |

*Connecticut.*

| | |
|---|---|
| William W. Rodman, | Agency of the sun and moon in periodicity. |

*New York.*

| | |
|---|---|
| Samuel Gordon Bailey, | On Prescribing. |
| Thomas Fitch, | On the Pelvis. |

*New Jersey.*

| | |
|---|---|
| George W. Allen, | Acute Hepatitis. |
| John W. Barcroft, | Influence of the Mind. |
| Nelson Burr, | Dysentery. |
| Samuel F. Fisler, | Obstipatio. |
| John B. Gilman, | Intermittent Fever. |
| James Risley, | Inflammation of the mucous membrane of the stomach. |
| A. Dickinson Woodruff, | Remittent Fever. |
| Granville S. Woolman, | Practical Obstetrics. |

*Pennsylvania.*

| | |
|---|---|
| James Rush Anderson, | Diagnosis of Typhoid Fever. |
| Elisha J. Baily, | Dysentery. |
| Wilson Baily, | Puerperal Fever. |
| Peter G. Bertolette, | Hygroma Patellæ. |
| Charles H. Bressler, | Influence of the Teeth on health. |
| George W. Brown, | Inflammation. |
| William H. Burr, | Cynanche Trachealis. |
| Archibald B. Campbell, | The heart and its functions as an evidence of design. |
| John S. Carpenter, | Puerperal Convulsions. |
| Henry T. Child, | Scrofula. |
| John Conrad, | Cystitis. |
| John Cox, | Neuralgia. |
| Edward Cronin, Jr. | Scarlatina. |
| Albert S. Cummings, | Diseases of the Heart. |
| Gordon Z. Dimock, | Acute Endocarditis. |
| Thomas W. Drake, | On Inebriating Liquors. |
| William P. Esrey, | Nicotiana Tabacum. |
| Josiah Haines, | Hernia. |
| William H. Hanly, | Acute Peritonitis. |
| Abraham Harshberger, | Internal Hernia. |
| Daniel Henderson, | Hæmoptysis. |
| James S. Hill, | Typhoid Fever. |
| John R. Hoskins, | Bloodletting. |
| Joseph Hannon, | Intermittent Fever. |
| Samuel B. Irwin, | Pneumonia. |
| Samuel Keneagy, | Animal Magnetism. |
| Philip H. Lang, | Asphyxia. |
| John Martin, | On Physiology. |
| Joseph Moyer, | Puerperal Fever. |
| Joseph M. McClure, | Scarlatina. |
| Daniel L. F. Oatman, | Intermittent Fever. |
| Adrian V. B. Orr, | Scarlatina. |
| Edward Parrish, | Cataract. |
| Albert Pearson, | Dysenteria. |
| William A. Piper, | Epidemic disease of Buffalo valley, in 1843. |
| Francis B. Poley, | Discrepancy of medical testimony. |
| John R. Quinan, | Origin, progress and present state of Medicine. |
| John S. Spriggs, | Sedative and febrifuge effects of Antim. et Pot. Tart. |
| George King Smith, | Comparative mortality of measles and Scarlet Fever. |
| James Steuart, | Erysipelas. |
| William W. Townsend, | Asthma. |
| Daniel A. Ulrich, | Anatomy of the Skin. |
| Tracy E. Waller, | Dysentery. |
| Alexander Wilcocks, | The Larynx and its functions. |
| Aaron Winder, | Manustupratio. |
| John D. White, | Treatment of exposed dental pulp. |
| Jacob L. Ziegler, | Puerperal Fever. |
| Samuel Morton Zulick, | Gonorrhœa. |

*Delaware.*

| | |
|---|---|
| Joseph P. Colgan, | Abnormal Anatomy of the fœtus. |
| Louis W. Hayes, | Defectio Animi. |

*Maryland.*

| | |
|---|---|
| Edward M. Hardcastle, | Tight Lacing. |
| Reginald N. Wright, | Rubeola. |

*Virginia.*

| | |
|---|---|
| John S. Bayn, | Scrofula. |
| James M. Barclay, | Mesmerism. |
| Thomas J. Buffington, | Physical Diagnosis in diseases of the heart. |
| Samuel Harris, | Acute Pleurisy. |
| William Travis Howard, | Pneumonia. |
| William R. Johnston, | Iodine. |
| John E. Moore, | Febris Intermittens. |
| Thomas J. Owen, | Rubeola. |
| William B. Paxton, | Influence of smoking on the mind. |
| Robert A. Phelps, | Emesis. |
| William T. Prentis, | Human Entozoa. |
| John P. Tabb, | Causes of Death. |
| William Upshaw, | Variola. |
| Michael Wallace, | Apoplexy. |
| Samuel E. Wills, | Anasarca. |

*North Carolina.*

| | |
|---|---|
| Richard B. Haywood, | Craniotomy. |
| George H. Mitchell, | Laryngitis Membranacea. |
| Thomas B. Powell, | Cynanche Trachealis. |
| William E. Wood, | Eclectic treatment of fever. |

*South Carolina.*

| | |
|---|---|
| William Furse, | Dyspepsia. |
| Benjamin W. Seabrook, | Dyspepsia. |
| William James Woods, | Acute Hepatitis. |

*Georgia.*

| | |
|---|---|
| Reuben S. Callaway, | Albuminuria. |
| William W. Durham, | Amenorrhœa. |
| Henry H. King, | Cholera Infantum. |
| N. Watkins Riddle, | Puerperal Fever. |

*Tennessee.*

| | |
|---|---|
| Plummer W. Dawson, | Remittent Fever. |

*Alabama.*

| | |
|---|---|
| William L. Antony, | Yellow Fever. |
| Courtney J. Clark, M.D. | Use of cold water in Fevers. |
| John F. Miller, | Coxalgia. |

*Mississippi.*

| | |
|---|---|
| Samuel Emanuel, | Remittent Fever. |
| Zachariah B. J. Griffing, | Pleurisy. |
| George H. Thornhill, | Ultimate Fibres. |

*Kentucky.*

| | |
|---|---|
| Amzi Martin, | Primary Syphilis. |

*Ohio.*

| | |
|---|---|
| Harvey C. Johnes, | Monstrosity. |
| John S. Kuhn, | Erysipelas. |

*Indiana.*

| | |
|---|---|
| Benjamin A. Allison, | Abuse of Cathartics. |
| James Darwin Maxwell, | Rubeola. |
| William P. Sunderland, | Milk Sickness. |
| Charles S. Weever, | Typhoid Fever. |

*Michigan.*

| | |
|---|---|
| Edmund G. Desnoyers, | De Hymene. |

*St. Thomas, W. I.*

| | |
|---|---|
| Baron J. F. Von Bretton, | On Regimen. |

Azariah B. Shipman, M. D., of New York, was admitted to the ad eundem degree of Doctor of Medicine in this Institution ; and the Honorary Degree of Doctor of Medicine was conferred on Dr. Isaac Winters and Dr. Rolph C. Marsh, of Pennsylvania. TOTAL, 117.

R. M. HUSTON, M. D., *Dean of the Faculty.*

